# TRPM8 inhibition reduces the size but increases the number of lipid droplets in mature adipocytes in vitro

**DOI:** 10.1016/j.jlr.2025.100935

**Published:** 2025-11-05

**Authors:** Parnasree Mahapatra, Shamit Kumar, Bisakha Das, Tusar Kanta Acharya, Chandan Goswami

**Affiliations:** 1 National Institute of Science Education and Research, School of Biological Sciences, Khurda, Odisha, India; 2 Homi Bhabha National Institute, Training School Complex, Mumbai, India

**Keywords:** mitochondria, Ca^2+^-buffering, cold, actin, cytoskeleton, metabolism, fission of LDs

## Abstract

Understanding the processes and/or the key factors involved in the formation as well as degradation of lipid droplets (LDs) within the adipocytes is of immense importance, especially in the context of health, obesity, cancer, and other diseases. While cold temperature and/or menthol (an edible cooling agent), seem to have diverse and confounding effects on obesity and/or thermogenesis, so far there is no direct evidence that specific pharmacological modulation of the Transient Receptor Potential cation channel subfamily Melastatin member 8 (TRPM8), a cold-temperature-activated ion channel, can indeed affect LD status within the mature adipocytes. Here, we used highly specific antagonists and agonists of TRPM8 to modulate TRPM8 in cultured adipocyte cells in vitro and monitored the expression of TRPM8 as well as other adipogenic functions. Our results indicate that specific activation of TRPM8 in mature adipocytes by a specific agonist, that is, WS12 ((1R∗,2S∗)-*N*-(4-methoxyphenyl)-5-methyl-2-(1-methylethyl)cyclohexanecarboxamide), results in increased expression of PPARγ protein. However, TRPM8 inhibition by *N*-(3-aminopropyl)-2-[(3-methylphenyl)methoxy]-*N*-(2-thienylmethyl)benzamidehydrochloride results in no change in the PPARγ expression, yet causes decreased Oil Red O intensity, a reduction in LD sizes, and an increase in LD numbers. BODIPY (4,4-difluoro-1,3,5,7,8-pentamethyl-4-bora-3a,4a-diaza-s-indacene) labeling in live cells also suggests the same findings. Altogether, data suggest that in the absence of any confounding factors, specific inhibition of TRPM8 results in either less fusion of LDs or enhanced fragmentation of LDs in vitro. These findings may have broad implications in the field of adipogenesis and in cancer.

## Graphical abstract

Schematic representation of simultaneous changes in cytosolic and mitochondrial Ca^2+^ in different conditions of adipogenesis. Mitochondrial Ca^2+^ (red box) is partly dependent on the cytosolic Ca^2+^ (green box) (represented by actual *r* values and also by flexibility, i.e., the ratio of mito/cyto Ca^2+^ in log10 scale; log-fold changes are indicated by numbers of ∗). Cytosolic Ca^2+^ and mitochondrial Ca^2+^ are represented in green and red filling, respectively. Cytosolic Ca^2+^ level is influenced by the net Ca^2+^ exchange of the cell with the extracellular environment and Ca^2+^ buffering by mitochondria (and also by other organelles). Ca^2+^ exchange is indicated by bidirectional arrows, and reduced exchange is indicated by short and dotted arrow. During AMTB treatment in induced condition, both cytosolic and mitochondrial Ca^2+^ levels become very high, and the correlation as well as flexibility between these two compartments becomes the lowest. Long-term AMTB treatment causes progressive loss (∼30%, ∼80%, and ∼90%) of cells in preadipocytes, differentiated adipocytes, and also in mature adipocytes, respectively. This could also be one of the prime reasons related to more LD fragmentation and/or inhibition of LD fusion in mature adipocytes.

**Figure:**
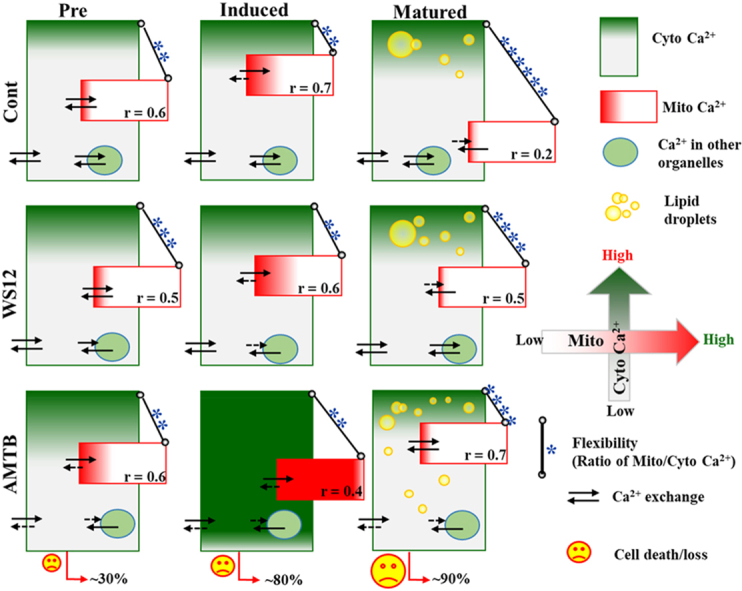

Adipogenesis is a complex and multifactorial process that is relevant to many pathophysiological conditions (1). Adipogenesis is relevant for energy storage, metabolism, heat generation, maintenance of body temperature, and many other essential physiological processes (2). In contrast, abnormalities in adipogenesis are related to obesity, cancer, inflammation, aging, hormonal imbalance, impaired immunity, and so on (3). Adipogenesis is the cellular mechanism for the production of lipid droplets (LDs), fat-laden subcellular organelles within cells with adipogenic potential. Adipogenesis is also influenced by complex behaviors, such as food uptake, stress, anxiety, depression, addiction, medication, age, etc (3, 4, 5). Among many external factors, both “hot” and “cold” temperatures are known to modulate adipogenesis (1). Accordingly, the administration of cooling agents, such as menthol and icilin, and heating agents, such as capsaicin, is known to modulate adipogenesis in vivo (6). Yet, the exact mechanism and/or factors involved in temperature, especially cold temperature- and/or cooling agent-induced adipogenesis, remain unexplored and/or debatable.

Adipogenesis is mainly due to the formation of LDs by the mature adipocytes, which involves complex Ca^2+^-signaling, changes in the expression of transcription factors, mitochondrial parameters, and many others. LDs are evolutionarily conserved organelles for energy storage and lipid transportation to the plasma membrane (7). Most of the triacylglycerol and cholesteryl esters are stored in LDs and are enclosed by a phospholipid monolayer (8).

A few members of the transient receptor potential subfamily are both thermosensitive and Ca^2+^-permeable ion channels, and these are involved in adipogenic differentiation (9, 10). Transient receptor potential cation channel subfamily melastatin member 8 (TRPM8) is a nonselective and the “true cold-activated channel” that allows a cytoplasmic influx of monovalent and divalent cations, including Ca^2+^ (11, 12). TRPM8 is nonspecifically activated by cooling agents, such as menthol and icilin, and is activated by temperatures lower than <25°C (11, 13, 14). TRPM8 is known to be expressed in human white adipocytes, mouse brown adipose tissue (BAT), and the murine preadipocyte cell line 3T3L1 (15, 16, 17). TRPM8 poses an important molecular target for understanding the mechanism as well as controlling adipogenesis.

So far, a few works have been performed with menthol (as a cooling agent) and/or TRPM8 (as a molecular target) in the context of adipocyte differentiation, functions, and/or thermogenesis, though the outcome of all these works remains largely contradictory and/or debatable. This is for several reasons. First, results obtained due to application of menthol (often as high as in millimolar concentration) cannot be considered as equivalent to TRPM8 activation exclusively, primarily as menthol can activate a series of other receptors and ion channels nonspecifically (such as voltage-gated sodium channels, voltage-gated potassium channels, TRP channels, gamma-aminobutyric acid and glycine receptors, nicotinic acetylcholine receptors, etc.), which all belong to diverse groups (18, 19, 20, 21, 22, 23). Second, the published literature suggests a “broad-range” of variations in menthol application, such as diverse routes (menthol-containing food uptake, direct administration to cell culture), doses, and regimes. Also, the relative changes (or no changes) in the expression of TRPM8, PPARγ, UCP1, and the browning as well as thermogenesis do not match in these conditions. Recently, we showed that menthol has more direct effects on mitochondria, as it cools mitochondria in different cells and experimental conditions (24).

Differentiation of human mesenchymal stem cells to adipocytes in response to menthol is also reported (25). TRPM8 is also detected in differentiated adipocytes and brown adipocytes (17, 26). Using TRPM8 knockout animals (*Trpm8*^*−/−*^) and immunoblotting as tools, it has been shown that TRPM8 is present in brown adipocytes, both in vivo as well as in cultured conditions (17). Expression of TRPM8-specific mRNA changes in mouse white adipocytes and also in predifferentiated and differentiated adipocytes (16). It has been shown that menthol-mediated TRPM8 activation increases thermogenesis (16). TRPM8 activation by menthol increases thermogenesis via thermogenic protein phosphorylation (16). Menthol supplementation with food induces fat browning, which leads to weight loss in obese animals maintained on a high-fat diet (16). However, no changes in the expression of TRPM8 are observed in the case of differentiation of white adipocytes (16). Both TRPM8 and PPARγ expression levels remain unchanged in different doses of menthol application (16). Another recent study also indicated that menthol application in the diet reduces body weight in the case of a diet-induced obese mouse model (27). They also showed that specific deletion of TRPM8 in neurons only causes obesity in mice that become glucose intolerant (27). All these reports impose an urgent need to reinvestigate the exact role of TRPM8 in adipocytes in the absence of any other confounding factors and with activators and inhibitors that are highly specific for TRPM8.

For this purpose, we have used the murine adipocyte cell line (3T3L1) in vitro, all in pre-, induced-, and differentiated stages, both in the absence as well as in the presence of TRPM8 modulators. Our data indicate that TRPM8 plays a complex role in the remodeling of the actin cytoskeleton, regulation of cytosolic and mitochondrial Ca^2+^-signaling, and the size and numbers of LDs.

## Materials and methods

### Preadipocyte culture and differentiation

The mouse fibroblast cell line 3T3L1 with adipogenic potential (obtained from NCCS, India) was cultured in DMEM (Gibco) supplemented with 10,000 units/ml of Penicillin, 10,000 μg/ml of Streptomycin, and 25 μg/ml of Amphotericin B (Gibco) and maintanied within cell culture incubator (5% CO_2_, 37°C). Cells were passaged by trypsin-EDTA (Sigma) and subsequently seeded on coverslips in either 6-well (for live cell imaging), 12-well (for fixed cell imaging), or 48-well (for MTT assay) plates. Cells before adding differentiation media were considered as “preadipocytes.” After cells reached ∼80–90% confluence, differentiation media were added, and cells were kept for differentiation for 3 days for preadipocyte differentiation to adipocyte cells. The differentiation media contained 3-isobutyl-1-methylxanthine (0.5 mM), dexamethasone (0.25 μM), rosiglitazone (2 μM), and insulin (5 μg/ml). Generally, 2 ml of differentiation media was added to 6-well plates/well. After 1 day of differentiation, 1 ml of preconditioned differentiation media was replaced with fresh differentiation media (in the case of 6-well plates). Once the cells were differentiated, maintenance media (insulin only, 5 μg/ml) was added for another 3–4 days. Generally, 2 ml of maintenance media was added to 6-well plates/well. Cells were routinely checked under the bright-field microscope, and bright LDs were observed after 8–10 days of adipogenic differentiation. TRPM8 activator WS12 ((1R∗,2S∗)-*N*-(4-methoxyphenyl)-5-methyl-2-(1-methylethyl)cyclohexanecarboxamide; (5 μM) and TRPM8 inhibitor *N*-(3-aminopropyl)-2-[(3-methylphenyl)methoxy]-*N*-(2-thienylmethyl)benzamidehydrochloride (AMTB) (10 μM) were used after 1 day of cell seeding, and the drug exposure is continued from preadipocyte up to adipocyte maturation, if not mentioned otherwise.

### Oil Red O staining

At first, mature adipocytes were fixed with 4% paraformaldehyde and washed with 1X PBS three times. Subsequently, cells were quickly rinsed with 60% isopropanol, and cells were incubated with filtered Oil Red O (ORO; HiMedia) solution made up with 60% isopropanol and autoclaved distilled water for 2 h. Then cells were washed with 1X PBS three times and incubated with 4',6-diamidino-2-phenylindole in 1X PBS for the next 10 min. Subsequently, cells were washed with 1X PBS three times, and coverslips containing mature adipocytes were mounted with Fluoromount-G (Southern Biotech) on glass slides.

### BODIPY staining

Cellular LDs were labeled by a neutral lipid-binding dye BODIPY 493/503 (4,4-difluoro-1,3,5,7,8-pentamethyl-4-bora-3a,4a-diaza-s-indacene) (Invitrogen). Both the untreated and WS12, AMTB-treated mature adipocytes were incubated with BODIPY (working concentration = 1.8 μM) for 30 min at 37°C. Live cell imaging of LDs was performed by an Olympus FV3000 confocal microscope.

### Immunofluorescence staining

Cells were first seeded on 18 mm coverslips, and all three stages (pre, differentiated, and mature) were maintained with the media, required supplements, and/or specific TRPM8 modulators. Cells were fixed with 4% paraformaldehyde at room temperature. Then cells are washed with 1X PBS twice and treated with 0.1% Triton X-100 (5 min). Subsequently, cells were washed with 1X PBS and incubated with BSA (for 1 h at room temperature). The cells were again washed with 1X PBS and then incubated with primary antibody (anti-TRPM8 [Alomone]) at 1:500 dilution and kept overnight at 4°C. Cells were washed and incubated with Alexa-Fluor 488 (1:500 dilution; Abcam) and kept for 1 h. For F-actin labeling, cells were further washed with 1X PBS and incubated with Phalloidin conjugated with Alexa-Fluor 594 (1:500 dilution; Invitrogen). Labeled cells were then washed with 1X PBS and mounted with Fluoromount G. The nucleus was stained with 4',6-diamidino-2-phenylindole (1:1,000 dilution; Invitrogen). For PPARγ staining, anti-rabbit monoclonal antibody as primary antibody (1:500 dilution; Cell Signaling Technology) and anti-rabbit secondary antibody conjugated with Alexa-Fluor 488 (1:500 dilution; Abcam) were used, and the same protocol as TRPM8 immunostaining was followed.

### Instant Ca^2+^-imaging

For instant Ca^2+^-imaging, 3T3L1 cells were grown on a glass-bottom dish and incubated with Fluo-4, AM dye (working concentration: 1 μM) for 1 h at 37°C. Live cell imaging was done with an Olympus FV3000 confocal microscope using an attached live cell chamber (Okolab) supplied with 5% CO_2_ and temperature maintained at 37°C. In some experimental conditions, cells were pretreated with AMTB (10 μM for 30 min). Cells were imaged for a total of 200 frames (total duration was 200 s), and WS12 was added at the 20th time frame. The fluorescence images were subjected to qualitative and quantitative analysis using ImageJ (Fiji/ImageJ) software.

### Live cell Ca^2+^-imaging

For measurement of cytosolic and mitochondrial Ca^2+^-levels, we used Fluo-4 AM (Invitrogen; excitation/emission: 494/506 nm) and Rhod-2 AM dye (Invitrogen; excitation/emission: 552/581 nm), respectively. Cytosolic and mitochondrial Ca^2+^ were measured separately or simultaneously as per the experimental requirements. Cells were incubated with Rhod-2 AM (5 μM) and/or Fluo4 AM (1 μM) for 1 h within the incubator maintained at 5% CO_2_ and 37°C. Media with excess dyes were discarded, and fresh media were added with TRPM8 activator and inhibitor. Live cell images were acquired by an Olympus FV3000 confocal microscope attached to a live cell chamber (Okolab) (5% CO_2_, 37°C).

### Image analysis

All the confocal microscopy images had been quantified and analyzed by ImageJ (Fiji) software. A minimum of 300 cells per experiment from three experimental repeats were quantified. Cellular parameters were analyzed by estimating the fluorophore intensity from the region of interest drawn manually. For LD quantification, digitally 4X zoomed images of cells were taken from different view fields, imaged with a 63× oil immersion objective to minimize background noise, and >20 cells were quantified. Individual LDs were marked as a single region of interest and counted and analyzed both manually and using particle analysis.

### MTT assay

The MTT assay was performed to check the cell survival of adipocytes in the presence of TRPM8 modulators in pre, differentiated, and matured conditions. For the MTT assay, 0.25 mg/ml of MTT solution was used. Briefly, in a 48-well plate, 3T3L1 cells (∼5 × 10^3^ cells/well) were seeded and treated with WS12 and AMTB in a humidified CO_2_ incubator at 37°C at different stages of adipogenesis. The cells were incubated for 4 h with the MTT dye. After the incubation, the MTT dissolving (SDS, 0.2%; HCl and isopropanol, 4 mM) reagent was added to every well and further incubated in the dark at 37 °C for 1 h. The absorbance values were acquired at 570 nm by using an ELISA plate reader (ThermoFisher).

### RNA isolation, RT, and quantitative real-time PCR

Total RNA was extracted from mature adipocytes in control, WS12-, and AMTB-treated conditions using the TRIzol method. RNA was extracted by TRIzol reagent (Invitrogen) as per the manufacturer’s protocol. Isolated RNA (1 μg) was reverse transcribed by the Verso Complementary DNA synthesis kit (Invitrogen) as per the manufacturer’s protocol, and gene expression was profiled by the quantitative real-time PCR method using SYBR Green master mix on the QuantStudio 7 Flex Real-Time PCR System (Applied Biosystems). For the quantitative PCR, 1 μg of complementary DNA was used as a template in all experimental conditions. The relative expression of PPARγ in all experimental conditions was normalized to the housekeeping gene GAPDH, and relative expression was calculated using the 2^-ΔΔCt^ method. These experiments were done in triplicate, and at least three biological replicates were used for the analysis. Primer sequences for GAPDH: forward (FP) 5′CATCACTGCCACCCAGAAGACTG3′, reverse (RP) 5′-ATGCCAGTGAGCTTCCCGTTCAG-3', PPARγ: forward (FP) 5′-CAGTTGATTTCTCCAGCATTTC-3′, reverse (RP) 5′-CTTTGATCGCACTTTGGTATTC-3′.

### Statistical analysis

All the data are expressed with the SEM. All the statistical analyses were done using ordinary one-way ANOVA, two-way ANOVA, and also paired *t* test by using GraphPad Prism, version 10.3. And *P* value <0.05 was considered statistically significant. Tukey multiple comparison test has been performed after ANOVA and *t*-test as post hoc analysis.

## Results

### Functional TRPM8 is present in 3T3L1 murine preadipocytes

**Fig 1:**
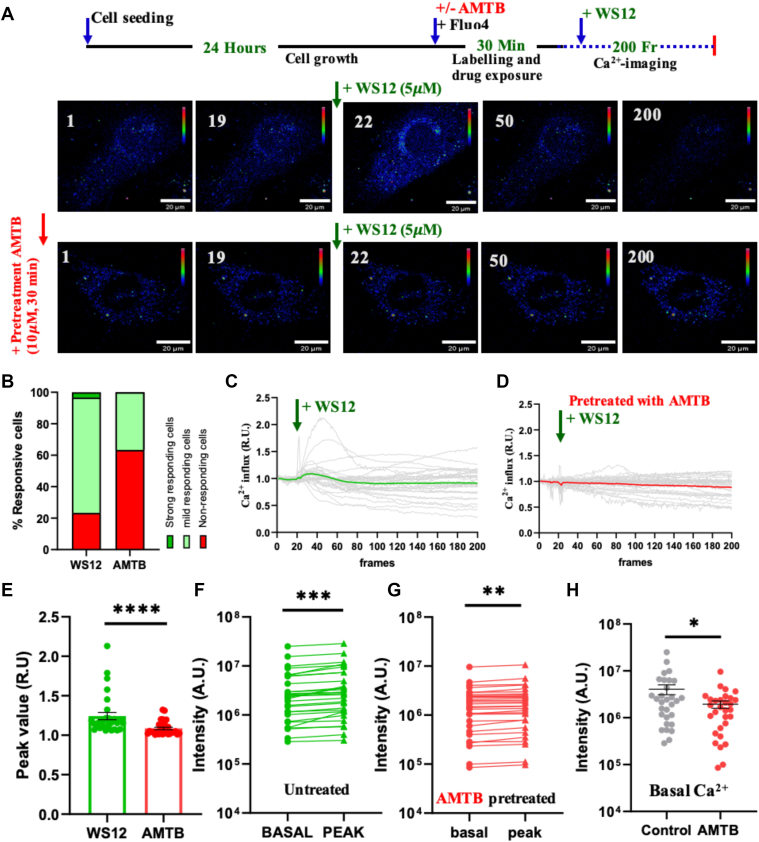
TRPM8 activation results in an increased Ca^2+^ in adipocytes. A: The experimental scheme and the time frames are shown at the top. Shown are the examples of time series Ca^2+^-imaging of control cells (top panel) or AMTB-pretreated cells (lower panel) that are treated with WS12 at the 20th frame. The fluorescence intensity of Fluo4, AM is shown in pseudo rainbow color. TRPM8 activation results in a modest but immediate increase in cytosolic Ca^2+^-level. B: The percentage of cells that are responding (dark green), mildly responding (light green), or not responding (red) in two different experimental conditions is shown. C, D: The changes in the cytosolic Ca^2+^-levels (in arbitrary units) of multiple cells are plotted with respect to time. All values are normalized (considered as 1 AU) to the basal value at the first frame. E: The peak values (in relative units) of cells are plotted. F and G: The basal cytosolic Ca^2+^-level and peak values of individual cells are compared in untreated cells (control, F) and AMTB-treated cells (10 μM for 30 min, G). For statistical analysis, the paired *t*-test has been performed. H: The cytosolic Ca^2+^ at the basal level (in arbitrary units) is shown for control as well as AMTB-pretreated cells. Multiple comparisons were performed by using a paired *t*-test. The statistical values are ns, nonsignificant, ∗ *P* < 0.05, ∗∗*P* < 0.01, ∗∗∗*P* < 0.001, and ∗∗∗∗*P* < 0.0001.

In this work, we used the 3T3L1 murine fibroblast cell line as a model system to study adipogenesis and the impact of pharmacological modulation of TRPM8 in vitro. In order to understand the relevance of TRPM8, we explored the functional expression of TRPM8 in 3T3L1. For that, 3T3L1 preadipocytes were labeled with a cytosolic Ca^2+^-sensor Fluo-4, AM and activated with WS12, a specific activator of TRPM8. Activation with WS12 results in a modest but immediate increase in the Ca^2+^-level in the responding population (Fig. 1A). In contrast, similar activation of the cells with WS12 but in the presence of AMTB (an inhibitor of TRPM8) results in a significant reduction in the cells that respond to the activation (Fig. 1A). Activation of TRPM8 suggests not all, but ∼77% of cells respond to the activation, and remaining cells are either not responding or may respond later (Fig. 1B). In contrast, AMTB-pretreated cells show ∼36% response against application of WS12. To get an estimation of WS12-mediated changes, we analyzed the basal Ca^2+^-level with respect to time (Fig. 1C). We also analyzed the peak value of the cells (Fig. 1D). WS12-treated cells show higher peak values as compared with the AMTB-pretreated cells (Fig. 1D). The effect of WS12-mediated activation was compared in control cells and also in AMTB-pretreated cells (Fig. 1E–F). The basal Ca^2+^-level shows significant reduction when treated with AMTB for 30 min (Fig. 1G). This suggests the importance of endogenous TRPM8 in the regulation of endogenous Ca^2+^-level in adipocytes.

### TRPM8 inhibition by AMTB induces cell death in 3T3L1

**Fig 2:**
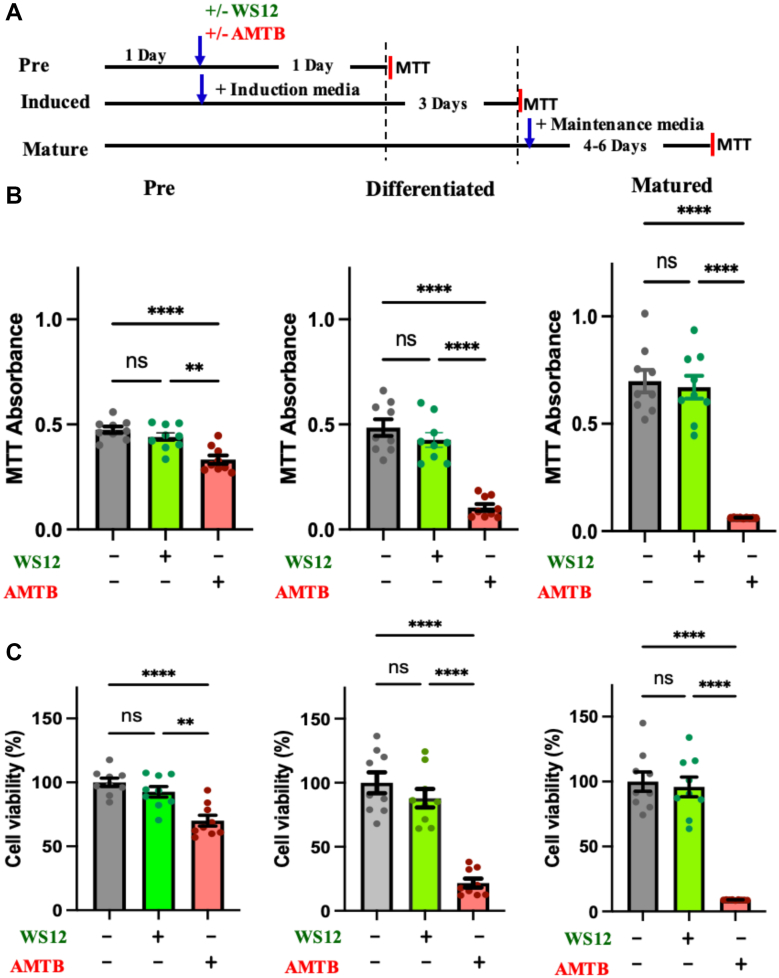
TRPM8 inhibition by AMTB results in progressive loss of adipocytes. A: The experimental scheme is shown. B: Shown are the results of the MTT assay of untreated (control) or treated with WS12 or AMTB in preadipocyte, differentiated adipocyte, and mature adipocyte 3T3L1 cells. C: The cell viability in different experimental conditions is shown. All values are normalized to the control condition (considered as 100%). Tukey multiple comparison test was performed after the one-way ANOVA. The statistical values are ns, nonsignificant, ∗∗*P* < 0.01, and ∗∗∗∗*P* < 0.0001.

We explored the functionality of TRPM8-specific modulators on 3T3L1 cells. Therefore, we treated the cells with WS12 (5 μM) and AMTB (10 μM) and performed the MTT assay for all the stages of maturations, that is, in preadipocytes, differentiations, and also maturation. We noted that both activation and inhibition of TRPM8 cause reduction in cell viability. But the reduction in cell viability is prominent in the case of TRPM8 inhibition (Fig. 2). In the AMTB-treated condition, only ∼70%, ∼21%, and ∼9% cells remain viable in pre-, induced-, and matured conditions. This suggests that endogenous TRPM8 function is critical for adipocyte maturation, and inhibition of TRPM8 activity results in progressive loss of adipocytes. The exact reason for this cell death is not clear, but Ca^2+^-overload in the cytosol as well as in the mitochondria is observed. This may suggest improper Ca^2+^-buffering because of TRPM8 inhibition.

### Endogenous TRPM8 and F-actin status vary during adipogenesis and in a context-dependent manner in vitro

**Fig 3:**
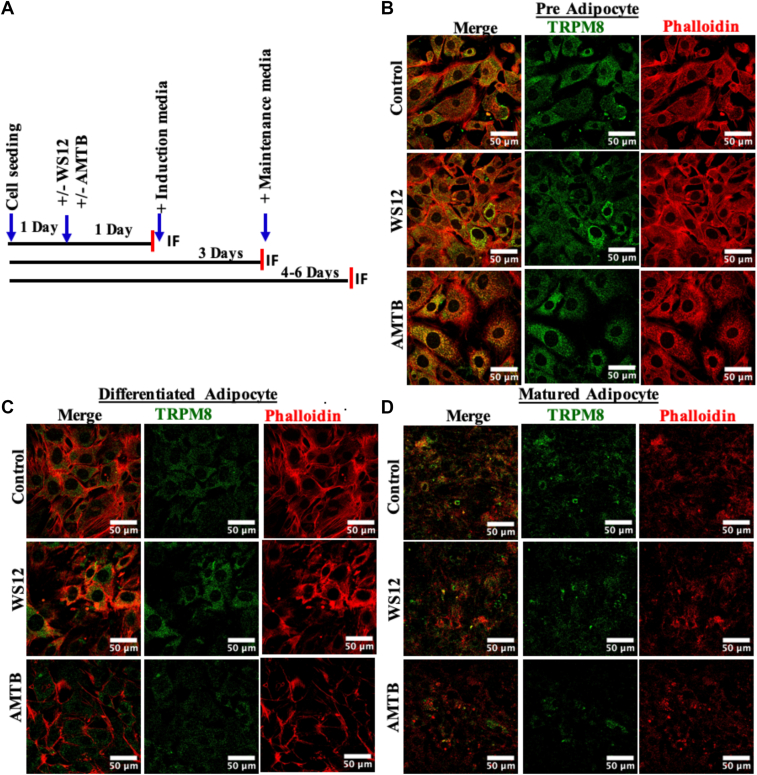
Expression of TRPM8 and F-actin changes in preadipocytes, differentiated adipocytes, and mature adipocytes. A: Murine fibroblast cell line 3T3L1 from a single stock is cultured and differentiated to mature adipocytes as described in the scheme. B–D: Immunostaining of the cells with anti-TRPM8 antibody (green) and Phalloidin staining for F-actin (red) are shown. Expression of TRPM8 and F-actin is significantly decreased in mature adipocytes that remain attached to the glass coverslips. F-actin becomes irregular and distorted in mature adipocytes as compared with pre- or differentiated conditions in these cells.

We analyzed the endogenous expression of TRPM8 in three different stages of adipogenesis (i.e., pre-, differentiated-, and matured-adipocytes), both in the absence and in the presence of TRPM8 modulators (WS12 as an activator and AMTB as an inhibitor) (Fig. 3A). As cellular stress-fiber levels have an impact on adipogenesis and LD formation in the mature adipocyte (28), we also analyzed the status and intensities of F-actin by probing with Phalloidin in the same experimental conditions. Endogenous TRPM8 and F-actin levels have been analyzed by confocal microscopy after immunostaining with anti-TRPM8 antibody and Alexa-Fluor 594-conjugated Phalloidin in all these stages and conditions (Fig. 3B–D). Both TRPM8 and patterns of stress fiber alter in these stages and/or because of TRPM8 modulations.

**Fig 4:**
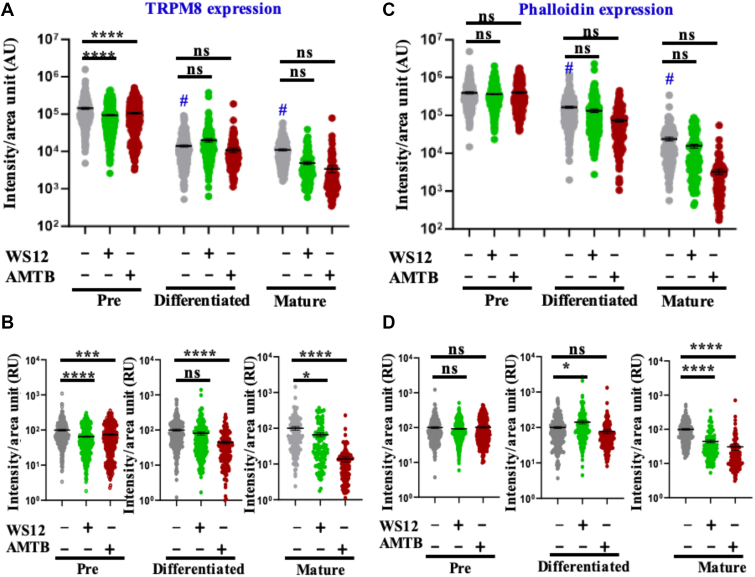
TRPM8 modulation affects both TRPM8 expression and F-actin levels. Experimental cells described in Fig 3 are quantified. A: Absolute values show that in both differentiated and mature conditions, there is no change in TRPM8 expression. In preadipocytes, TRPM8 expression decreases both in WS12- (activator) and AMTB- (inhibitor) treated conditions. B: In different stages of adipogenesis, the relative changes in TRPM8 are seen. TRPM8 expression significantly decreases in AMTB-treated conditions in mature adipocytes, as compared with the control condition. Also in preadipocytes, TRPM8 expression decreases in WS12- and AMTB-treated conditions as compared with the control. C: Absolute fluorescence values depict mostly nonsignificant changes in F-actin levels throughout the adipogenesis differentiation. D: The relative intensity of F-actin significantly decreases in AMTB-treated conditions in mature adipocytes. For both TRPM8 and F-actin, multiple comparisons were performed by using one-way ANOVA (for B and D) and two-way ANOVA (for A and C), and multiple comparisons after one-way/two-way ANOVA were analyzed by Tukey multiple comparison tests. The statistical values are ns, nonsignificant, ∗*P* < 0.05, ∗∗*P* < 0.01, ∗∗∗*P* < 001, and ∗∗∗∗*P* < 0.0001. #TRPM8 expression or F-actin level changes in control of differentiated and mature adipocytes in comparison to preadipocyte control.

Next, we quantified a large number of cells from multiple sets and compared the expression levels, both in an absolute manner as well as in a relative manner. We observed that in the absence of any TRPM8 modulators, the endogenous TRPM8 expression significantly decreases but remains detectable in differentiated and matured adipocytes in comparison to preadipocytes (Fig. 4A). However, in mature adipocytes, both TRPM8 activation and inhibition cause further reduction (though nonsignificantly) in TRPM8 levels. However, when compared in a "relative manner" (i.e., considering the expression of TRPM8 in the absence of any modulators as a control condition, 100%), the normalized expression of TRPM8 varies significantly in preadipocytes and mature adipocytes in response to TRPM8 modulations (Fig. 4B). In the case of mature adipocytes, TRPM8 expression significantly decreases in response to WS12 or AMTB in comparison to the control (Fig. 4B).

Changes in actin dynamics are significant phenomena in adipogenesis. In the absence of any TRPM8 modulators, overall the levels of stress fibers (as labeled by Phalloidin) become significantly low in differentiated and in matured adipocytes in comparison to preadipocytes (Fig. 4C). Insufficient stress fiber may affect the cell adhesion, and this can also be a reason for progressive loss of cells during adipocyte maturation.

However, in a relative comparison, inhibition of TRPM8 leads to a significant reduction in F-actin level, both in induced and matured adipocytes, but not in preadipocytes when compared with the control condition (Fig. 4D). In contrast, activation of TRPM8 shows nonsignificant changes in F-actin levels in preadipocytes and induced adipocytes but shows a reduction in matured adipocytes (Fig. 4D).

### Long-term modulation of TRPM8 causes different levels of cytosolic Ca^2+^

**Fig 5:**
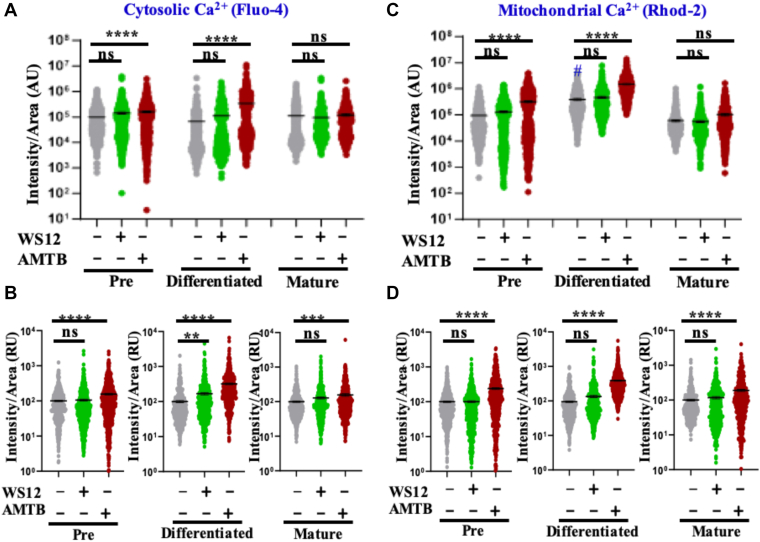
Inhibition of TRPM8 increases overall mitochondrial and cytosolic Ca^2+^-levels in preadipocytes, induced adipocytes, and mature adipocytes. A: The absolute fluorescence intensity of cytosolic Ca^2+^ from the preadipocytes to the differentiated adipocytes is shown. Only in AMTB-treated conditions in differentiated adipocytes did total cytoplasmic Ca^2+^ increase. All absolute values are plotted in arbitrary units (AUs). B: The relative fluorescence intensity of cytosolic Ca^2+^ in all stages of cell differentiation is plotted, and in all cases, the average values of the control condition are considered as 100%. Notably, in the TRPM8-inhibited condition, the cytosolic Ca^2+^-level increases in all conditions. Absolute values plotted above (A) are used for this relative calculation. C: The absolute fluorescence intensity of mitochondrial Ca^2+^ from the preadipocytes to the differentiated adipocytes is shown. An increment in mitochondrial Ca^2+^-level is seen in AMTB-treated conditions, both in pre- and differentiated adipocytes. D: The relative fluorescence intensity of mitochondrial Ca^2+^ in all stages of cell differentiation is plotted, and in all cases, the average values of the control condition are considered as 100%. Absolute values plotted above (C) are used for this relative calculation. In AMTB-treated conditions, mitochondrial Ca^2+^ increases. In all cases, the statistical values were obtained by one-way ANOVA (for B and D) and two-way ANOVA (for A and C), and multiple comparisons after one-way/two-way ANOVA were analyzed by Tukey multiple comparison tests. ns, nonsignificant, ∗*P* < 0.05, ∗∗*P* < 0.01, ∗∗∗*P* < .001, and ∗∗∗∗*P* < 0.0001. # indicates significant changes in comparison to the control condition of preadipocytes. At least 100 cells/condition and a total of three biological replicates are quantified (N = 3) for each condition.

As TRPM8 is a Ca^2+^-permeable ion channel, the overall cytosolic Ca^2+^ has been measured using Fluo4 AM. We have observed nonsignificant changes in the Fluo4 AM intensities in preadipocytes, induced adipocytes, and mature adipocytes both in the presence and absence of any modulators (Fig. 5A). However, only in the differentiated conditions (but not in preadipocyte and mature adipocyte), inhibition of TRPM8 shows a significant increment in cytosolic Ca^2+^-levels (Fig. 5A). However, upon comparing the cytosolic Ca^2+^ in a "relative" manner, we observed a significant rise in cytosolic Ca^2+^ in the case of TRPM8 inhibition by AMTB (Fig. 5B). This may suggest a change in cytosolic Ca^2+^-buffering because of inhibition of TRPM8. Notably, TRPM8 activation does not lead to a further increase in cytosolic Ca^2+^ in preadipocytes as well as in mature adipocytes but causes a significant increase in induced adipocytes (Fig. 5B).

### Long-term modulation of TRPM8 causes different levels of mitochondrial Ca^2+^

We measured the mitochondrial Ca^2+^-levels in these cells during adipogenesis in the absence as well as in the presence of TRPM8 modulators. Mitochondrial Ca^2+^ is significantly increased in induced adipocytes but not in matured adipocytes as compared with preadipocytes (Fig. 5C). In TRPM8-inhibited condition, mitochondrial Ca^2+^-level increases in preadipocytes and induced adipocytes but not in mature adipocytes (Fig. 5C). However, on a “relative” comparison, TRPM8 inhibition, but not activation, shows increased mitochondrial Ca^2+^ as compared with the control in all these stages (Fig. 5D).

### Long-term modulation of TRPM8 alters the relationship of mitochondrial Ca^2+^ to cytosolic Ca^2+^

**Fig 6:**
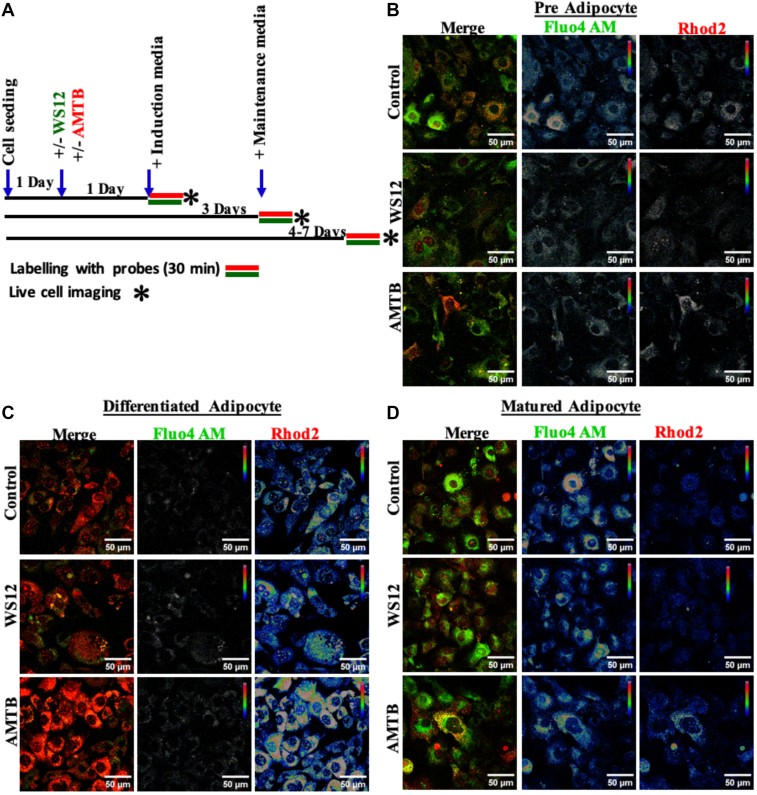
Simultaneous detection of cytosolic and mitochondrial Ca^2+^ in different experimental conditions and in differentiation stages. A: Cellular Ca^2+^-level is measured by Fluo4 AM (green), and mitochondrial Ca^2+^ is measured by Rhod-2 AM (red) simultaneously in different experimental conditions. B–D: Merged images are provided in the leftmost, and the corresponding images depict the fluorescence intensities of Fluo4 AM and Rhod-2 AM dyes in a pseudo-rainbow scale (red and blue indicate the highest and the lowest signal, respectively).

**Fig 7:**
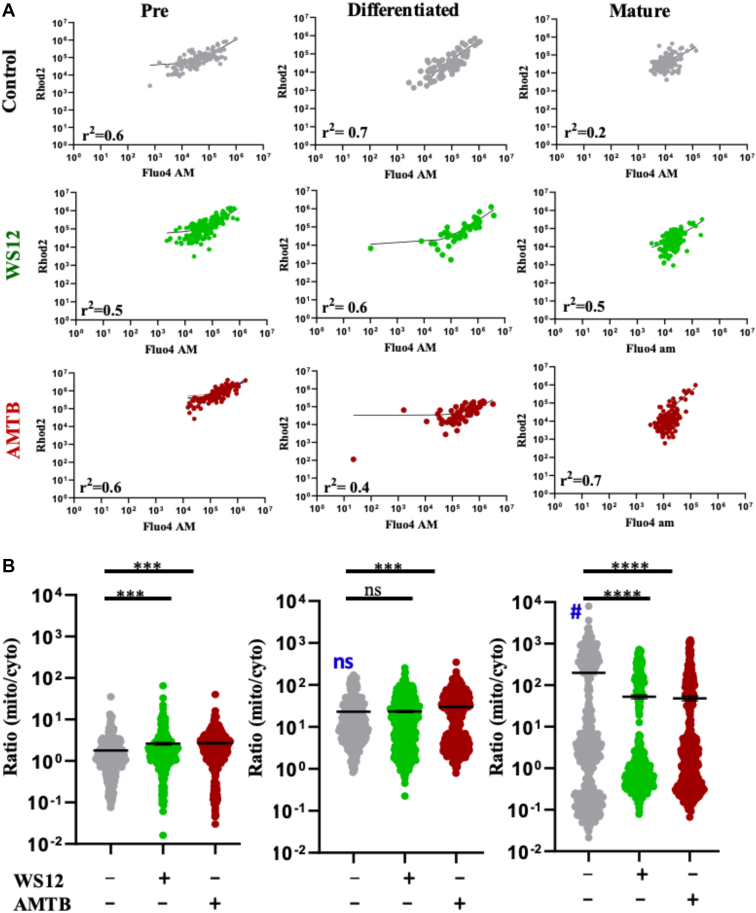
Dependency of mitochondrial Ca^2+^ on cytosolic Ca^2+^ changes during adipogenic differentiation and pharmacological modulation of TRPM8. A: Shown is the correlation of mitochondrial Ca^2+^ (values from Rhod-2 AM) with respect to cytosolic Ca^2+^ (Fluo4 AM) in different experimental conditions. In all cases, the absolute fluorescence values are plotted. In mature adipocytes, TRPM8 inhibition causes mitochondrial Ca^2+^ to be more dependent on cytosolic Ca^2+^. B: The ratio of mitochondrial Ca^2+^ with cytosolic Ca^2+^ in different stages and in experimental conditions is shown. Absolute fluorescence values of mitochondrial Ca^2+^ and cytosolic Ca^2+^ were used to analyze the ratio. Statistical values were obtained by performing one-way ANOVA, and multiple comparisons after one-way ANOVA were analyzed by Tukey multiple comparison tests. ns, nonsignificant, ∗∗∗*P* < 001, ∗∗∗∗*P* < 0.0001. # indicates significant changes in comparison to the control condition of preadipocytes. At least 100 cells/set and a total of three sets of cells are quantified (N = 3) for each condition.

To analyze the relationship of mitochondrial Ca^2+^ to cytosolic Ca^2+^, we detected mitochondrial Ca^2+^ to cytosolic Ca^2+^ simultaneously in all these different experimental conditions and all these differentiation stages (Fig. 6). We also analyzed the correlation of these two parameters in different conditions (Fig. 7A). The correlation values fluctuate from 0.4 to 0.7 in all different conditions, suggesting that the mitochondrial Ca^2+^ remains partially dependent on the cytosolic Ca^2+^ and/or modest Ca^2+^-buffering by mitochondria. However, in mature adipocytes, the correlation value becomes lowest (*r* = 0.2) suggesting that in this control condition, the mitochondrial Ca^2+^-buffering ability probably becomes very low. In preadipocytes, the correlation value was rescued (*r* = 0.5) in the case of TRPM8 activation, but the value became highest in the case of TRPM8 inhibition by AMTB (*r* = 0.7). These comparatives suggest that in mature adipocytes, TRPM8 inhibition converts mitochondrial Ca^2+^ highly dependent on the cytosolic Ca^2+^, possibly suggesting improved Ca^2+^-buffering by mitochondria.

We also analyzed the ratio of mito-Ca^2+^/cyto-Ca^2+^ in different conditions (Fig. 7B). In general, in the absence of any modulators, the average ratio values (mito-Ca^2+^/cyto-Ca^2+^) increase during adipogenic differentiation. Accordingly, the average value remains low (0.79) in the case of preadipocytes, modest (23.12) in induced adipocytes, and highest (197.61) in mature adipocytes. Notably, this "fluctuation" range of the ratio remains within ∼100-fold in preadipocytes (varies from 10^-1^ to 10^1^) and induced adipocytes (varies from 10^0^ to 10^2^). But the same "fluctuation" becomes very high (∼1,000,000) in the case of mature adipocytes (varies from 10^-2^ to 10^4^). This high fluctuation may indicate that in all these stages, the relationship of mitochondrial Ca^2+^ with respect to cytosolic Ca^2+^ is probably regulated differently. This ratio increases in the case of TRPM8 activation (1.45-fold) as well as inhibition (1.49-fold) in the case of preadipocytes. In the case of induced adipocytes, TRPM8 inhibition increases this ratio (1.29-fold) but not by activation. Notably, in mature adipocytes, both activation (0.26-fold) and inhibition (0.24-fold) of TRPM8 cause a reduction of the ratio.

### TRPM8 activation, but not the inhibition, increases PPARγ expression

**Fig 8:**
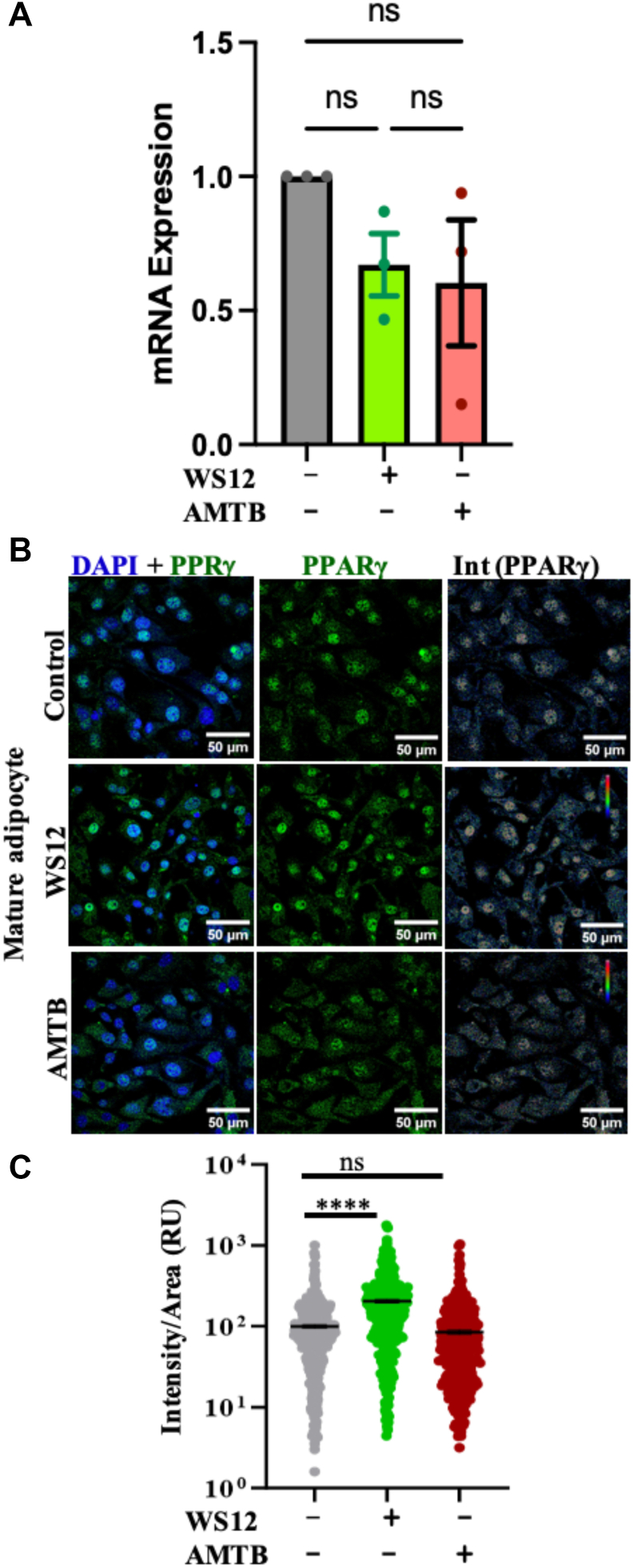
TRPM8 activation but not inhibition of TRPM8 increases PPAR-γ expression in mature adipocytes. A: mRNA expression of PPARγ in control, activated, and inhibited conditions of mature adipocytes is compared. B: Shown are the confocal images of mature adipocytes treated with or without TRPM8-modulatory agents for at least 10 days, fixed, and further labeled with anti-PPAR-γ antibody (green) and nucleus with DAPI (blue). C: The quantitative representation of PPARγ expression per cell in control and TRPM8-modulated conditions is shown. The average expression value of PPARγ in the control condition is considered as 100%, and the expression in other conditions was normalized accordingly. Statistical significance test: one-way ANOVA and multiple comparisons after one-way ANOVA were analyzed by Tukey multiple comparison tests. ns, nonsignificant, ∗∗∗∗*P* < 0.0001. At least 100 cells/set and a total of three sets of cells are quantified (N = 3) for each condition. DAPI, 4',6-diamidino-2-phenylindole.

Next, we analyzed the expression of PPARγ levels, both in mRNA and protein forms in the matured adipocytes, as PPARγ is a nuclear receptor subfamily protein for different transcription factors, which are involved in adipogenic differentiation and maturation (29). At the RNA level, both TRPM8 activation and inhibition cause reduction in PPARγ levels (though data remain statistically nonsignificant for treated conditions compared with control) (Fig. 8A). However, we noted that TRPM8 activation by WS12 but not TRPM8 inhibition by AMTB results in higher expression of PPARγ in the protein level, at least in the cells that remain attached to the glass coverslips (Fig. 8B). To confirm the same, we quantified the PPARγ expression per cell from multiple cells and noted that TRPM8 activation caused a significant increase (∼2-fold) in the expression of PPARγ. TRPM8 inhibition by AMTB does not have any effect on the expression of PPARγ.

### TRPM8 inhibition reduces the size but increases the number of LDs

**Fig 9:**
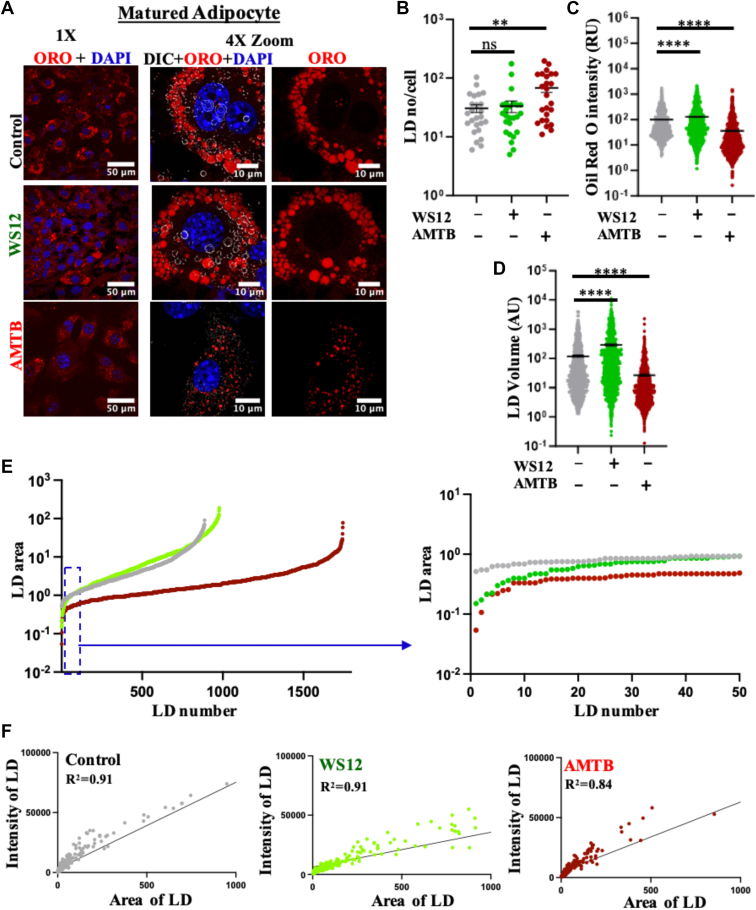
TRPM8-specific inhibitor AMTB reduces the LD size and volume but increases the number of mature adipocytes. A: Confocal images of mature adipocytes labeled with ORO (red, for detecting LDs) and DAPI (blue, for DNA) in control as well as in WS12- or AMTB-treated conditions are shown. In general, TRPM8 activation induces bigger LDs, and inhibition causes smaller LDs. B: TRPM8 inhibition increases the number of LDs per cell. At least 25 cells were quantified in each condition. C: The fluorescence intensity of ORO per cell is quantified in different conditions. The average quantity of ORO in the control condition is considered 100%, and the values from other conditions were normalized accordingly. TRPM8 inhibition reduces the fluorescence of ORO labeling. D: TRPM8 modulations with WS12 and AMTB alter the LD volume in matured adipocytes. LD volume increases in the case of TRPM8 activation but decreases in the case of TRPM8 inhibition. Fluorescence intensities as well as the area of individual LD from at least 25 cells from three biological replicates in each experimental group are presented. Statistical significance: one-way ANOVA and multiple comparisons after one-way ANOVA were analyzed by Tukey multiple comparison tests. ns, nonsignificant, ∗∗*P* < 0.01, ∗∗∗∗*P* < 0.0001. E: Analysis of LD sizes in different conditions. Individual sizes of all detectable LDs were arranged in an ascending order. TRPM8 inhibition reduces the sizes but increases the total number of LDs. The size distribution of the lowest 50 LDs in different experimental conditions was shown. F: Correlation analysis of the fluorescence intensity with the area of the individual LDs in different experimental conditions is plotted. DAPI, 4',6-diamidino-2-phenylindole.

To analyze the LD status, we performed ORO staining of mature adipocytes in the absence as well as in the presence of TRPM8 modulators. The imaging of mature adipocytes (that remain adherent to glass coverslip) suggests that the intensity of ORO increases in WS12-treated conditions and decreases in AMTB-treated conditions as compared with control (Fig. 9A). Next, we quantified the number of LDs per cell. There is a significant increase observed in the number of LDs in the case of TRPM8 inhibition as compared with the control (Fig. 9B). However, there is no change in the number of LDs in the case of TRPM8 activation (Fig. 9B). Further, the total fluorescence intensity of ORO per cell is quantified in these different conditions. The fluorescence intensity is significantly increased (2.9-fold) in the case of TRPM8 activation and significantly decreased (0.09-fold) in the case of TRPM8 inhibition (Fig. 9C). We also quantified the LD volumes in these conditions. The LD volume is increased (2-fold) in the case of TRPM8 activation and decreased (0.22-fold) in the case of TRPM8 inhibition (Fig. 9D).

To evaluate the size distribution of all these LDs in different conditions, we arranged all the detected LDs in an ascending order of size. We noted that TRPM8 inhibition causes an increased number of LDs, yet with smaller sizes as compared with the control or TRPM8-activated condition. Thus, inhibition may cause more "fragmentation" of the bigger LDs and/or less fusion of the smaller LDs. In order to test that, we compared the smallest 50 LDs from 25 cells in control, TRPM8-activated, and TRPM8-inhibited conditions. We noted the presence of the smallest LDs in the TRPM8-inhibited condition but not in the control or TRPM8-activated condition (Fig. 9E). We also analyzed the correlation between the fluorescence intensity of ORO with the individual LD volumes in all these three experimental conditions. In all these conditions, the fluorescence intensity correlates well with the LD volumes (*R*^2^ values are as follows: control: 0.91, WS12: 0.91, and AMTB: 0.84). All these data suggest that TRPM8 inhibition prevents fusion of smaller LDs and/or induces fragmentation of bigger LDs (discussed later).

**Fig 10:**
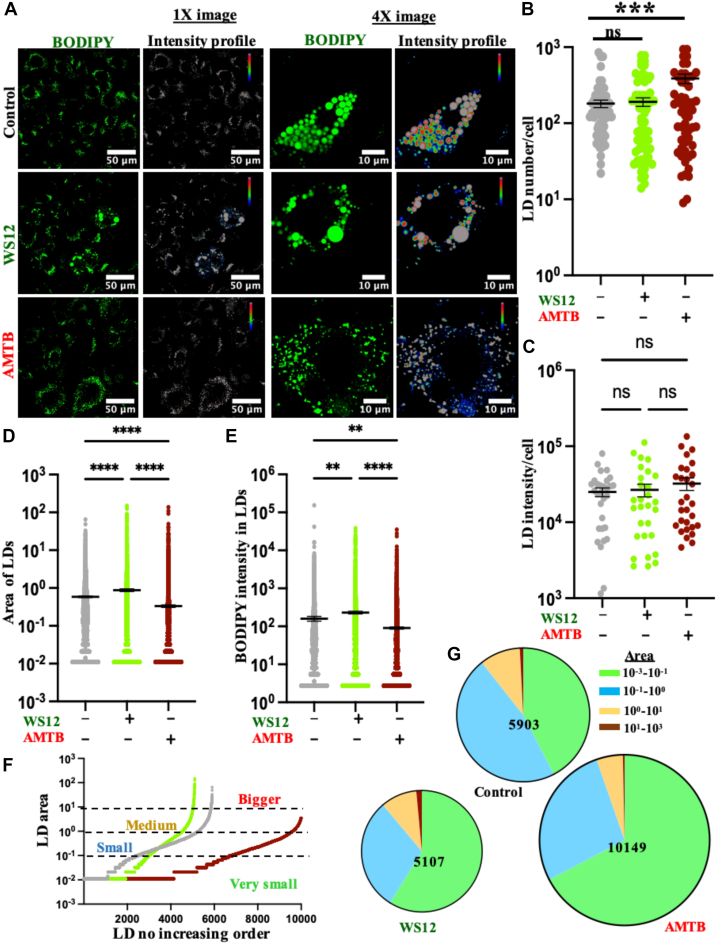
TRPM8-specific inhibitor AMTB increases the number of LDs in live mature adipocytes. A: Confocal images of live mature adipocytes labeled with BODIPY (green) in control, WS12-, and AMTB-treated conditions are shown. In general, TRPM8 inhibition induces a greater number of smaller LDs. B: TRPM8 inhibition increases the number of LDs per cell. At least 25 cells were quantified in each condition. C: The fluorescence intensity of BODIPY per cell is quantified in different conditions. D and E: Individual LDs were quantified from 30 cells in each experimental condition from three biological replicates and plotted. The area (D) and fluorescence intensity (E) of individual LDs are plotted. Statistical significance: one-way ANOVA and multiple comparisons after one-way ANOVA were analyzed by Tukey multiple comparison tests. ns, nonsignificant, ∗∗*P* < 0.01, ∗∗∗∗*P* < 0.0001. F: Analysis of LD sizes in different conditions. Individual sizes of all detectable LDs were arranged in an ascending order. TRPM8 inhibition increases the total number of LDs, most of which are smaller in size. G: Pie chart demonstrating the percentage distribution of very smaller (green), smaller (blue), medium (yellow), and bigger (maroon) LDs in different experimental conditions. The size of the pie circle is adjusted as per the total number of LDs detected in control, WS12-, and AMTB-treated conditions.

ORO staining is not very sensitive and may suffer from the cell fixation procedure. Therefore, to analyze the effect of TRPM8 modulation on the LD by another independent method, we labeled the live cells with BODIPY. In accordance with the ORO labeling, BODIPY labeling in live cells also indicates that TRPM8 inhibition by AMTB results in fragmentation of LDs (Fig. 10A). The number of LDs per cell also increased in the case of AMTB treatment (Fig. 10B). Total fluorescence intensity of BODIPY per cell remains nonsignificantly different in WS12- and AMTB-treated conditions, at least in the cells that remain attached to the coverslips (Fig. 10C). Analysis of individual LD volume as well as fluorescence intensity indicates that an increased number of smaller LDs are absent in the case of AMTB-treated conditions (Fig. 10D–E). Arrangement of LDs (as size in 2D) in ascending order also supports the same (Fig. 10F). We classified the LDs as very smaller (10^-3^–10^-1^ μm^2^), smaller (10^-1^–10^0^ μm^2^), medium (10^0^–10^1^ μm^2^), and bigger (10^1^–10^3^ μm^2^) as per the size. Analysis of the percentage of LDs indicates that in case of AMTB, the bigger LDs are low, but smaller and very smaller LDs are higher in percentage (Fig. 10G). Collectively, the data indicate that AMTB induces smaller-sized LDs in greater numbers.

## Discussion

### Thermosensitive TRP channels including TRPM8 are relevant for the regulations of LD formation

**Fig 11:**
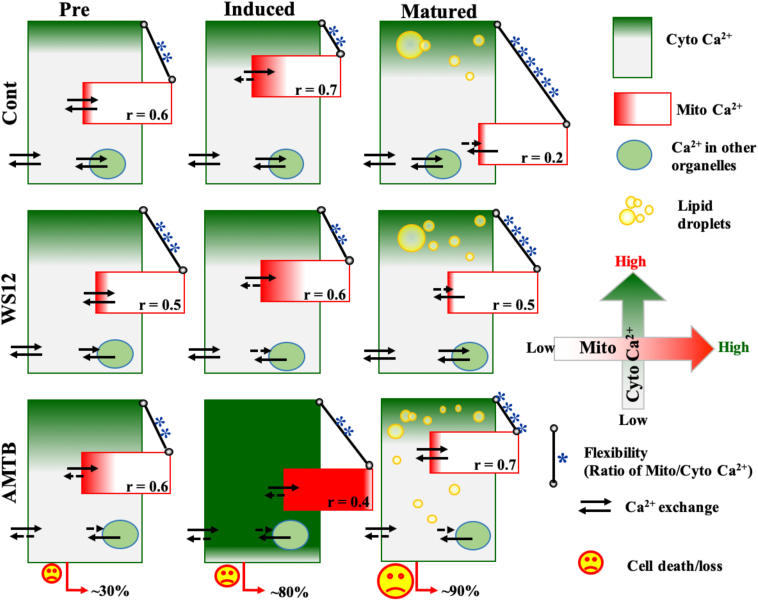
Schematic representation of simultaneous changes in cytosolic and mitochondrial Ca^2+^ in different conditions of adipogenesis. Mitochondrial Ca^2+^ (red box) is partly dependent on the cytosolic Ca^2+^ (green box) (represented by actual *r* values and also by flexibility, i.e., the ratio of mito/cyto Ca^2+^ in log10 scale, log-fold changes are indicated by numbers of ∗). Cytosolic Ca^2+^ and mitochondrial Ca^2+^ are represented in green and red filling, respectively. Cytosolic Ca^2+^-level is influenced by the net Ca^2+^-exchange of the cell with the extracellular environment and Ca^2+^-buffering by mitochondria (and also by other organelles). Ca^2+^-exchange is indicated by bidirectional arrows; reduced exchange is indicated by short and dotted arrows. AMTB treatment in induced condition, both cytosolic and mitochondrial Ca^2+^-levels become very high, and the correlation as well as flexibility between these two compartments becomes lowest. Long-term AMTB treatment causes progressive loss (∼30%, ∼80%, and ∼90%) of cells in pre, differentiated, and also in mature adipocytes, respectively. This could also be one of the prime reasons related to more LD fragmentation and/or inhibition of LD fusion in mature adipocytes.

In this work, using the 3T3L1 cell line as a model system and using different microscopic techniques, we show that in the mature stage, TRPM8 inhibition causes a significant reduction in ORO content but increases the number of LDs (Fig. 11). In this context, the outcome of inhibition of the cold-activated ion channel (TRPM8) is equivalent to the activation of the hot-activated ion channel (such as TRPV1 activation by capsaicin) (30). This is also in line with our recent observation that inhibition of TRPV4, another hot-activated ion channel, causes increased LD formation (31). Notably, AMTB-treatment results in progressive loss of cells, and up to 90% of cells are lost in the case of mature adipocytes. Therefore, the microscopic data are relevant for cells that remain attached to the coverslips.

### Effect of TRPM8 inhibition on LD dynamics

Notably, the size comparison of LDs (as detected by ORO as well as BODIPY labeling) suggests that AMTB-treated cells (i.e., in TRPM8-inhibited condition) have much smaller LDs. The lower 10% as well as the top 10% of the LDs (as labeled by ORO) are much smaller in the case of AMTB-treated cells when compared with control or even WS12-treated cells (data not shown). All these largely point out that TRPM8 inhibition either causes fragmentation of bigger LDs and/or inhibits fusion of smaller LDs, though the exact mechanism is still not clear. Involvement of enhanced cytosolic Ca^2+^ may also prevent the fusion of smaller LDs, a possibility that cannot be ruled out.

Interestingly, the LDs detected in ORO in fixed cells and BODIPY have certain differences. A population of smaller-sized LDs (especially at the range of 10^-1^ square μm or lower) is detected in the case of BODIPY, but such a population is missing in the case of ORO labeling in all the experimental conditions. This can be due to the differences in the cell labeling, as ORO labeling needs cell fixation and further extraction. On the other hand, BODIPY-labeled LDs are detected in live cell conditions.

There are several factors that seem to be changed or remain unaltered because of the long-term application of AMTB, which is a highly specific inhibitor of TRPM8 (32). Notably, another recently developed TRPM8 antagonist, that is, PF-05105679, is able to reverse the browning effect of menthol in human white adipose tissue in culture conditions (25). Thus, the data we present here with AMTB are different from the findings described by Goralczyk *et al*. However, the exact specificity of PF-05105679 remains questionable. Nevertheless, all these data endorse TRPM8 as a potential molecular target for the regulation of LDs within white adipocytes.

Here, we discuss different aspects that seem to be relevant for TRPM8-mediated regulation of LDs. For example, we observed no significant change in the expression of PPARγ in the case of long-term AMTB-treated conditions. Therefore, the reduction in LD volume and increase in size because of TRPM8 inhibition seem to be independent of changes in PPARγ expression. However, we noted a change in the status of F-actin. For commitment to LD-producing cells, fibroblast precursor cells follow the remodeling of actin filaments in order to accumulate LDs (33, 34, 35). Accordingly, the overall F-actin labeling is low in mature adipocytes, and in the case of AMTB-treatment, the level becomes the lowest. This is contradictory and unique to the general norm that actin depolymerization enhances LD formation (34).

### Possible involvement of Ca^2+^ in the regulation of LD biogenesis

In mature adipocytes, expression of TRPM8 is lower as compared with preadipocytes or induced adipocytes. Even in mature adipocytes, TRPM8 activation as well as inhibition further reduces the TRPM8 expression level but remains at detectable levels. We also measured the cytosolic and mitochondrial Ca^2+^-levels in individual cells and all these stages. This allowed us to analyze the relationship between mitochondrial Ca^2+^ and cytosolic Ca^2+^ (Fig. 8). We noted that in most of the conditions, the “*r* value" remains at a range of 0.5–0.6, suggesting the existence of a mitochondrial population that is partially dependent on the cytosolic Ca^2+^. However, there are conditions where this “*r* value" increases to 0.7, suggesting a condition where the mitochondrial population becomes more dependent on the cytosolic Ca^2+^. Notably, cytosolic Ca^2+^-level increases because of TRPM8 inhibition during all these stages (i.e., pre-, induced-, and mature) of adipogenesis. TRPM8 activation by WS12 does not induce many notable changes in cytosolic Ca^2+^, especially in preadipocytes and matured adipocytes. This in general suggests that endogenous TRPM8 may be involved in functions that are related to the lowering of cytosolic Ca^2+^ and/or cytosolic Ca^2+^-buffering by intracellular organelles. Indeed, our data suggest that TRPM8 seems to be involved in Ca^2+^-buffering by mitochondria. Mitochondrial Ca^2+^-level also increases significantly because of AMTB application in induced adipocytes and also to a certain extent in preadipocytes and matured adipocytes as compared with the respective control conditions. These data suggest a drastic change in both cytosolic and mitochondrial Ca^2+^-levels because of AMTB application in induced adipocytes rather than in mature adipocytes. In induced adipocytes, TRPM8 inhibition causes the highest amount of Ca^2+^ in the cytosol as well as in mitochondria. In the same condition, the dependency of mitochondrial Ca^2+^ on cytosolic Ca^2+^ is the least (*r* value <0.2), suggesting that in this condition, the mitochondrial population is much less correlated with cytoplasmic Ca^2+^, and thus, mitochondrial Ca^2+^-overloading is fully or partially independent of the cytosolic Ca^2+^-levels. All these also indirectly suggest that TRPM8 might be present in the mitochondria (and also in other intracellular organelles involved in cytosolic Ca^2+^-buffering) and regulate mitochondrial Ca^2+^^-^levels. Indeed, there is a 4TM version of TRPM8 that is reported to be present in the mitochondria (36).

### Possible cellular and molecular mechanisms for LD number regulation

LD size is maintained by various mechanisms, such as triacylglycerol storage, autophagy, lipolysis, fission-fusion, etc. (23, 37, 38, 39). LD fission leads to an increase in the number of LDs and maintains the total volume (equivalent to the total amount). In contrast, LD fusion leads to a decrease in total LD number, yet maintains the volume. LD fusion-fission is a tightly regulated mechanism of poorly understood events. Most of the SNARE protein assembly is a Ca^2+^-dependent process. Some previous research showed that LD fusion is involved in the SNARE-mediated process (38, 39, 40, 41) and the whole process can be greatly influenced by Ca^2+^-signaling. LD fusion in adipocytes is mediated by the CIDE family of proteins (42, 43). It is possible that high cytosolic Ca^2+^, high mitochondrial Ca^2+^, or both are relevant for the fragmentation of LDs as observed in AMTB-treated conditions, as in such conditions proper Ca^2+^-levels and signaling events can be achieved. Several hydrolytic enzymes are present in the cytosol, which can degrade LDs. Some of these lipases are known to require higher Ca^2+^-levels for their optimum functions (44, 45). Storage-operated Ca^2+^-influx in turn activates neutral lipases in the cytosol, which results in the reduction of LD size (42, 43, 44, 45). Large LDs have a small surface-to-volume ratio (also the surface curvature) as compared with the smaller LDs, where surface curvature is higher. Lipolysis events happen more efficiently in the case of smaller LDs, which will make them even smaller (46). Lipolytic fission promotes the exit of fatty acids from the LDs and leads to the lipase-mediated reduction in LD size. AMTB is reported as a potent therapeutic agent to suppress osteosarcoma, prostate cancer, and breast cancer (47, 48, 49).

### AMTB as a possible antiadipogenic compound

There are several facts that indeed suggest the efficacy of AMTB toward reduction of LD. Primarily, the lowest LDs detected (by ORO) in the case of AMTB-treated cells are smaller than the lowest LDs observed in other conditions. The “dose-response” experiment also suggests that at a 10 μM concentration of AMTB, there is a clear reduction in the LD droplet number per cell, LD area per cell, and LD intensity per area (data not shown). At lower concentrations, the effect is not clear. This indicates a complex and nonlinear relationship achieved at a 10 μM concentration of AMTB.

The correlation value (fluorescence intensity with area of individual LD) in different experimental conditions (control = 0.91, WS12 = 0.91, and AMTB = 0.84) shows a slight reduction in the *r*2 value for AMTB. This may suggest a “qualitative change” in the LDs that affect ORO-binding to the LDs. Data based on BODIPY, another probe used for detecting LD in live cells, also accord well with the Oil-Red-based data. Considering all these findings, our data suggest that TRPM8 inhibition by AMTB results in reduction of LD sizes. There might be certain signaling event(s) that seem to be triggered by TRPM8 inhibition at 10 μM concentration but not effectively at lower concentrations.

### Possible involvement of mitochondria in LD biogenesis alteration

So far, it is known that Mfn2 is critical for the BAT-mediated thermogenic function (50). According to their findings, specific knockout of Mfn2 from BAT results in bigger and a greater number of LD formations (50). In accordance with Boutant *et al*., another work has also proposed that adipocytes of obese animals have less Mfn2 (51). However, in a contradictory manner, Mann *et al*. (52) have proposed that loss of Mfn1, but not Mfn2, results in enhanced adipogenesis. Other mitochondrial fission and fission regulatory proteins, such as Opa1 and Drp1, also regulate the LD status and overall adipogenesis (53, 54, 55). Notably, other thermosensitive ion channels, like TRPV4, are present in the mitochondria, and TRPV4 interacts with Mfn2 and other mitochondrial proteins (56, 57, 58, 59, 60). Considering the TRPV4 as an example, TRPM8-mediated complex regulation of mitochondria is also possible.

### Limitations and conclusions

Though this work suggests that TRPM8 inhibition affects the survival of mature adipocytes, the exact molecular mechanism remains unknown. Also the detailed changes in the mitochondria are not fully understood. Also, the involvement of other subcellular organelles, like the endoplasmic reticulum and lysosome, needs further investigation. In this work, we show that TRPM8 inhibition by AMTB at 10 μm concentration lowers the bigger LDs and increases the number of smaller LDs. Our data suggest that long-term and localized inhibition of TRPM8 by AMTB can be useful for reducing LD volume and thus may prove useful for treating obesity. However, further in vivo work is needed.

## Data availability

Data will be made available on a reasonable request.

## Conflict of interest

The authors declare that they have no conflicts of interest with the contents of this article
